# Healthcare professionals' intentions and behaviours: A systematic review of studies based on social cognitive theories

**DOI:** 10.1186/1748-5908-3-36

**Published:** 2008-07-16

**Authors:** Gaston Godin, Ariane Bélanger-Gravel, Martin Eccles, Jeremy Grimshaw

**Affiliations:** 1 Canada Research Chair on Behaviour and Health, Laval University, Québec, Canada; 2 Research Group on Behaviour and Health, Faculty of Nursing, Laval University, Québec, Canada; 3 Institute of Health and Society, Newcastle University, Newcastle upon Tyne, UK; 4 Clinical Epidemiology Program, Ottawa Health Research Institute, Ontario, Canada; 5 Department of Medicine, University of Ottawa, Ontario, Canada

## Abstract

**Background:**

There is an important gap between the implications of clinical research evidence and the routine clinical practice of healthcare professionals. Because individual decisions are often central to adoption of a clinical-related behaviour, more information about the cognitive mechanisms underlying behaviours is needed to improve behaviour change interventions targeting healthcare professionals. The aim of this study was to systematically review the published scientific literature about factors influencing health professionals' behaviours based on social cognitive theories. These theories refer to theories where individual cognitions/thoughts are viewed as processes intervening between observable stimuli and responses in real world situations.

**Methods:**

We searched psycINFO, MEDLINE, EMBASE, CIHNAL, Index to theses, PROQUEST dissertations and theses and Current Contents for articles published in English only. We included studies that aimed to predict healthcare professionals' intentions and behaviours with a clear specification of relying on a social cognitive theory. Information on percent of explained variance (R^2^) was used to compute the overall frequency-weighted mean R^2 ^to evaluate the efficacy of prediction in several contexts and according to different methodological aspects. The cognitive factors most consistently associated with prediction of healthcare professionals' intention and behaviours were documented.

**Results:**

Seventy eight studies met the inclusion criteria. Among these studies, 72 provided information on the determinants of intention and 16 prospective studies provided information on the determinants of behaviour. The theory most often used as reference was the Theory of Reasoned Action (TRA) or its extension the Theory of Planned Behaviour (TPB). An overall frequency-weighted mean R^2 ^of 0.31 was observed for the prediction of behaviour; 0.59 for the prediction of intention. A number of moderators influenced the efficacy of prediction; frequency-weighted mean R^2 ^varied from 0.001 to 0.58 for behaviour and 0.19 to 0.81 for intention.

**Conclusion:**

Our results suggest that the TPB appears to be an appropriate theory to predict behaviour whereas other theories better capture the dynamic underlying intention. In addition, given the variations in efficacy of prediction, special care should be given to methodological issues, especially to better define the context of behaviour performance.

## Background

Healthcare professionals are continually exposed to new research findings that could contribute to more effective and efficient patient care. Unfortunately, the transfer of research findings into practice does not happen as readily as desired [1], and many authors have documented gaps between evidence-based practices and the routine clinical practice of healthcare professionals [2,3].

A wide range of factors can influence the clinical practice of healthcare professionals [4], including individual motivational predispositions to change as well as economic, political, and organizational contexts. However, our understanding of these factors and optimal approaches to change healthcare professional behaviour is incomplete. This has led to calls for more theory-based research to better inform the design of interventions to change healthcare professionals' behaviour [1,5,6]. Although several theoretical perspectives could be used to explore the determinants of the healthcare professionals' behaviours, most or many clinical practice adoption decisions are individual professional decisions [7]. Consequently, it would be useful to obtain a better understanding of the individual mechanisms of the adoption of new behaviours from social psychology theories [8]. For the purpose of this review, social cognitive theories refer to theories where individual cognitions/thoughts are viewed as processes intervening between observable stimuli and responses in real world situations.

The problem of understanding why healthcare professionals do or do not implement research findings can be viewed as similar to finding out why people in general do or do not adopt a given behaviour such as health-related habits. This has been extensively investigated, and social psychological theories have already demonstrated their value. For the prediction of health-related behaviours, there are several social cognitive theories that predict moderate to large amount of the variance of intention and behaviour [9].

It is surprising that relatively little attention has been given to reviewing published studies applying social cognitive theories investigating healthcare professional behaviours. It is only recently that two publications have reviewed specific aspects of theory-based studies of healthcare professional behaviour and practice. Eccles and colleagues [10] concluded that intention was a valid proxy measure for behaviour among clinicians (*i.e.*, physicians, nurses, pharmacists, other health workers). They did not quantify the strength of association between intention and behaviour among healthcare professionals, but based on the review of ten prospective studies, they concluded that this association was similar in magnitude to that reported for non-professional populations. For example, in a quantitative summary of meta-analyses, Sheeran estimated that, on average, 28% of the variance in behaviour (R^2^) is accounted for by intentions [11].

A review by Perkins and colleagues [12] was limited to applications of the theories of reasoned action (TRA) [13] and planned behaviour (TPB) [14] to understand clinicians' behaviour (*i.e. *physicians, nurses, pharmacists, other health workers). They found very few studies (N = 19), and only half of them (N = 9) included a measure of behaviour (eight self-reported; one objective from medical record). As in the review by Eccles and colleagues [10], they also did not quantify the strength of association between TRA/TPB constructs and actual behaviour, but nonetheless concluded that different constructs of these two theories predict intention and behaviour among different groups of clinicians.

Obviously, more information is needed regarding the usefulness of social cognitive theories to understand and predict healthcare professionals' intentions and behaviours. The aim of this study was to review systematically the literature to quantify to what extent studies based on social cognitive theories explain intention of healthcare professionals to adopt clinical behaviours and predict health professionals' clinical behaviour. Given that any of several social cognitive theories could have been used to investigate healthcare professional behaviours, this review was not limited to applications of the TRA and TPB. Other social theories such as Bandura's social cognitive theory [15], Triandis' theory of interpersonal behaviour [16] and others theories of behaviour were included as well.

## Methods

### Inclusion and exclusion criteria

We included studies that assessed the predictive value of clearly specified social cognitive theories (*e.g.*, theory of planned behaviour, social cognitive theory, theory of interpersonal behaviour, etc.) for clinician intentions and/or clinical behaviours. It must be mentioned that these theories are considered 'theories of the problem' (*i.e.*, determinants) instead of 'theories of the action' (*i.e.*, change). Clinical behaviours were defined as any behaviour performed in a clinical context. We only included prospective studies focusing on prediction of behaviour, *i.e.*, studies assessing behaviour at a later point in time following the assessment of the theoretical constructs; this was done in order to respect one of the main theoretical assumptions of the majority of the social cognitive theories [13,17]. Studies that predicted behaviour instead of intention within a cross-sectional design were excluded. However we did include cross-sectional studies focusing on prediction of intention. Finally, studies aimed at predicting students' behaviours (except for residents in medicine) were excluded because these were not considered clinical-related behaviours.

### Literature search

The literature search was performed between September 14 and October 30, 2007 by ABG. We searched psycINFO (1960–2007), MEDLINE (1966–2007), EMBASE (1974–2007), CIHNAL (1982–2007), Index to theses (1970–2007), PROQUEST dissertations & theses (1960–2007), and Current Contents (2006–2007) for articles published in English only. The search strategy was behaviour OR intention AND [health professionals] (see Additional file 1: The literature search). This was modified as appropriate for the other databases such as MEDLINE and EMBASE. ABG undertook the initial screen of the search results for potentially included studies. ABG and GG then screened potentially included studies against the inclusion criteria. For all included studies, the reference lists were checked manually.

### Review methods

Data about authors and year of publication, population under study, sample size, study design, main theory used, variable predicted (intention/behaviour), kind of behaviour, variables measured, and main results were abstracted by ABG and reviewed by GG; this is summarized in electronic tables (see Additional file 2: Prospective studies aimed at predicting health professionals' behaviour, and Additional file 3: Studies aimed at predicting health professionals' intentions). Duplicate data abstraction was undertaken for 15% of the dataset by SA. Disagreements were resolved by consensus between ABG, GG and SA. When necessary, we attempted to contact the authors by e-mail for key missing data elements.

Before analyzing the data set, a number of decisions were taken. First, several of the published studies used the same sample to predict different intentions/behaviours. In this situation, we selected at random one of the intention/behaviour models in order to avoid attributing more weight to such studies. Second, a few studies reported results from application of different theories to the same sample. For the same reason mentioned above, only the model with the highest explained variance was retained for analysis.

For the analysis, we calculated an overall frequency-weighted mean R^2 ^for intentions and behaviours. We also documented the variables measured and the number of times these variables contributed significantly (*p *< 0.05) to the prediction of the dependent variable (*i.e.*, variables most consistently associated with intention or behaviours). These variables were classified according to the theoretical domains defined by Michie and colleagues [8] (see Additional file 4: Classification of variables). However, in order to take into consideration the ethical dimension of healthcare professional behaviours, moral norm was retained as an additional category. Also, although past behaviour and habits are not psychosocial constructs *per se*, these two factors were retained as another category. In addition, we explored the impact of a number of *a priori *defined potential moderators by comparing the frequency-weighted mean R^2 ^for different categories of moderators using Fisher's Z transformation procedure for correlations. A small number of empirical criteria (*i.e.*, moderators) were used to evaluate the efficacy of the studies to predict intention/behaviour. Moderators included: type of professional (*e.g.*, physicians, nurses, pharmacists, etc.); type of behaviour (*e.g.*, prescribing, compliance with guidelines, wearing gloves, perform an examination, etc.); main theory used (*e.g.*, theory of planned behaviour, social cognitive theory, etc); sample size; psychometric qualities; type of dependent variable measurement (objective: direct observation, documentation from databases and behaviour reported from the patients; subjective: self-reported behaviour) and the level of correspondence between intention and behaviour. Based on the work of Rashidian and colleagues [18], we dichotomized the studies in two categories: less than 150 respondents versus 150 and more. For psychometric qualities, we dichotomized internal consistency as good (Cronbach's alpha coefficient of 0.60 or more) versus poor/no information provided [19]. If only partial information was provided, the studies were classified as 'good' if the reported psychometric qualities met the standards. The level of correspondence between intention and behaviour was evaluated according to Fishbein and Ajzen's guidelines [13]; that is, intention and behaviour must correspond in terms of action (*e.g.*, advise to have), target (*e.g.*, retina screening), context (*e.g.*, patients with type 2 diabetes), and time (*e.g.*, during the next three months). Studies for which the measurement of intention and behaviour corresponded in terms of action, target, and context were classified as having a good intention-behaviour level of correspondence; the time element was not considered.

## Results

### Description of included studies

**Figure 1:**
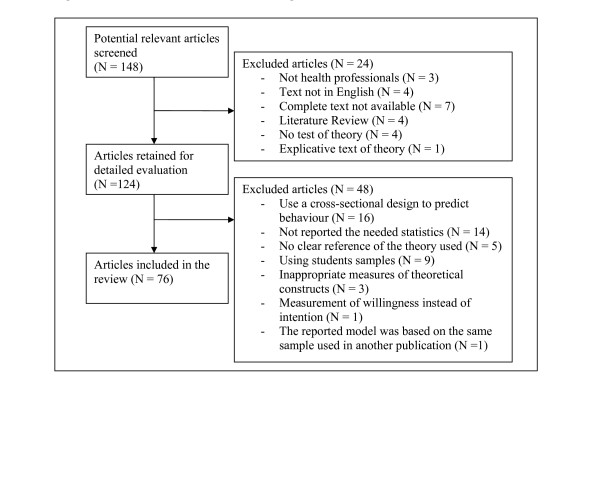
The QUORUM statement flow diagram.

Results from the bibliographic screen are presented in Figure 1. Seventy-six studies (N = 20,259 participants) were included in the review. Among these, 16 studies adopted a longitudinal design to predict healthcare professional's behaviours. In addition, 72 of these studies provided information on determinants of intention.

Clinical-related behaviours were investigated in populations of physicians [20-25], nurses [26-32], and other health professionals (*i.e.*, pharmacists [33,34] and psychologists [35]). Among physicians, the behaviours investigated were related to clinical practice (*e.g.*, prescribing, performing an examination, referring patients to specialists, etc.) [20-23], compliance with guidelines (*e.g.*, hand hygiene and wearing gloves) [24], and counseling [25]. Among nurses, the behaviours studied were related to clinical practice (*e.g.*, professional support for labour, pain management, providing care to patients, etc.) [26,30,31], compliance with guidelines [27,28], and documentation [29,32]. Clinical practice [35] and counseling [33,34] were also investigated for other professionals.

For the prediction of intention, several studies were also available for the different categories of health professionals: physicians [20,21,23-25,36-59], nurses [26-31,60-82], and other clinicians [35,83-95]. Other clinicians included pharmacists [85,88,90,94], dentists [83,95], mental health professionals [86,87], psychologists [35], social workers [91], and a mix of different professions [84,89,92,93]. Among studies of physicians' intention, the prediction of intention related to clinical practice (*e.g.*, prescribing, performing an examination, referring patients to specialists, etc.) [20,21,23,37,38,41,48,49,53-55,57-59], acceptance of technologies [40,42,45,46,51], compliance with guidelines (*e.g.*, hand hygiene and wearing gloves) [24,36,44,50,56], counseling [25,39,52], and documentation [43,47]. Among nurses, their intentions related to clinical practice (*e.g.*, professional support for labour, pain management, providing care to patients, etc.) [26,30,31,60-64,66-72,74-79,81], acceptance of technologies [65], compliance with guidelines [27,28,73,80,82], and documentation [29]. Clinical practice [35,83,84,87,91,95], compliance with guidelines [89,92,93], and counseling [85,86,88,90,94] were also investigated for other professionals.

### Social cognitive models efficacy

There were important variations in efficacy of prediction of behaviour and intention; the R^2 ^varied from 0.001 to 0.58 for behaviour and 0.14 to 0.91 for intention. Overall, the frequency-weighted mean R^2 ^for the prediction of behaviour was 0.31 (Number of studies (N) = 15, number of professionals (N) = 2,112) and 0.59 (N = 64, N = 14,986) for the prediction of intention. The overall efficacy of prediction according to the main theory used to guide the studies is presented in Table 1. For the prediction of behaviour, the theory most often used as reference was the TRA or its extension the TPB. Only one study used the operant learning theory (OLT) [96], and another one used the social cognitive theory (SCT) [15]. The predictive power of studies employing the TRA/TPB to predict health professionals' behaviours was significantly better than studies employing the other theories (Z = 6.085; *p *< 0.0001).

**Table 1:** Overall efficacy of prediction according to the theory used in the studies

Main theory used to model...	Number of participants (studies)	Frequency- weighted mean R^2^
Behaviour		
- Theory of planned behaviour (theory of reasoned action)	1,882 (14)	0.35
- Others*	230 (1)	0.06
		
Intention		
- Theory of interpersonal behaviour	734 (3)	0.81
- Theory of planned behaviour (theory of reasoned action)	13,188 (56)	0.59
- Technology acceptance model	535 (2)	0.47
- Others	529 (3)	0.42

Note: Because there were missing data in few publications, total differs from 16 and 72 studies for the behaviour and intention, respectively.

* Only the study based on the Operant Learning Theory was included; the other study did not provide information on R^2^.

For the prediction of intention, the theories most frequently used to guide the studies were, in order of importance, the TRA/TPB, the technology acceptance model (TAM) [97], the theory of interpersonal behaviour (TIB), the OLT and, finally, the attitude, social and self-efficacy model (ASE) [98]. However, among these theories, studies based on the TIB best predicted health professionals' intentions (Z = 12.461; *p *< 0.0001, Z = 11.287; *p *< 0.0001 and Z = 12.389; *p *< 0.0001 for the comparison with TPB/TRA, TAM, and the other theories, respectively).

### Most consistent variables associated with behaviour and intention

The number of times the variables were assessed and found to have a significant effect for the prediction of behaviour and intention is presented in Table 2. Among the variables assessed, the cognitive factors most consistently associated with prediction of healthcare professional's behaviours (*i.e.*, at least 50% of the time) were beliefs about capabilities (sample size-weighted average correlation: r_+ _= 0.18, k = 7, N = 1,484), and intention (sample size-weighted average correlation: r_+ _= 0.46, k = 11, N = 1,754). Beliefs about consequences, social influences, past behaviour, and knowledge were also reported to be correlates of behaviour, but to a lesser extent. The other variables were not assessed at least three times and no further analysis was performed.

**Table 2:** Variables measured and associated with behaviour and intention

Variables measured	Number of time		Ratio
		
Prediction of behaviour	Assessed	Significant (p < 0.05)	(Significant/assessed) × 100 (%)
Intention	12	6	50.0
Beliefs about consequences	9	4	44.4
Beliefs about capabilities	8	5	62.5
Social influences	6	2	33.3
Past behaviour	5	1	20.0
Knowledge	2	1	N/A
Role & identity	2	0	N/A
Moral norm	1	0	N/A
Emotion	1	0	N/A
Personal characteristics	1	1	N/A
Environmental factors	1	1	N/A
Prediction of intention			
Beliefs about consequences	79	58	73.4
Social influences	75	47	62.3
Beliefs about capabilities	65	51	78.5
Past behaviour	31	14	45.2
Characteristics of HP	29	11	37.9
Moral norm	14	10	71.4
Role & Identity	14	8	57.1
Emotion	9	3	33.3
Knowledge	8	1	12.5
Environment	4	1	25.0

N/A: not computed because it was not measured at least three times.

With respect to the factors explaining intention, the most consistently significant cognitive factors (*i.e.*, at least 50% of the time) were beliefs about capabilities, beliefs about consequences, moral norm, social influences, and social/professional role and identity. Other determinants frequently reported were past behaviour and emotion. Finally, the less frequently significant variables were socio-demographic characteristics, environmental influences, and knowledge.

### Type of professional and behaviour

The efficacy of the studies using social cognitive theories to explain intention and predict behaviour of healthcare professionals for different types of professionals and behaviours is presented in Table 3. The comparison of the computed frequency-weighted mean R^2 ^between healthcare professional categories indicated that compared to physicians and nurses' behaviours the prediction for other professionals was better (Z = -5.791; *p *< 0.0001 and Z = -6.069; *p *< 0.0001, respectively). For the prediction of intention, there were significant differences between the frequency-weighted mean R^2 ^values of all types of professionals (physicians versus nurses: Z = -13.414; *p *< 0.0001; physicians versus other professionals: Z = -5.909; *p *< 0.0001; and nurses versus other professionals: Z = 6.009; *p *< 0.0001) with the better prediction observed in studies of nurses.

**Table 3:** Model efficacy to predict healthcare professionals' behaviours and intentions according to the type of professional and behaviours

Healthcare professionals	Behaviour categories	Number of participants (studies)	Frequency- weighted mean R^2^*
Prediction of behaviour			
Physicians	Clinical practice	387 (4)	0.11
	Compliance with guidelines	33 (1)	0.001
	Counseling	765 (1)	0.40
	Total	1 185 (6)	0.28

Nurses	Clinical practice	220 (3)	0.41
	Compliance with guidelines	225 (2)	0.19
	Documentation	158 (2)	0.09
	Total	603 (7)	0.24

Other professionals	Clinical practice	284 (1)	0.58
	Counseling	40 (1)	0.33
	Total	324 (2)	0.55

Prediction of intention			
Physicians	Clinical practice	2 185 (11)	0.54
	Acceptance of technologies	1 150 (4)	0.68
	Compliance with guidelines	762 (4)	0.50
	Counseling	1 146 (3)	0.28
	Documentation	180 (2)	0.19
	Total	5 423 (24)	0.51

Nurses	Clinical practice	4 443 (21)	0.68
	Acceptance of technologies	151 (1)	0.77
	Compliance with guidelines	1 181 (5)	0.62
	Documentation	108 (1)	0.46
	Total	5 883 (28)	0.66

Other professionals	Clinical practice	2 042 (6)	0.53
	Compliance with guidelines	527 (1)	0.73
	Counseling	1 111 (5)	0.62
	Total	3 680 (12)	0.59

Note: Because there were missing data in few publications, total differs from 16 and 72 studies for the behaviour and intention, respectively.

### Methodological moderators of the efficacy of prediction

The efficacy of prediction of behaviour and intention according to different methodological moderators is presented in Table 4. The results indicate that the prediction of behaviour and intention was significantly better when sample sizes were equal to or greater than 150 participants compared to smaller samples (behaviour: Z = -4.710; *p *< 0.0001; intention: Z = -8.643; *p *< 0.0001). Concerning the psychometric qualities, no difference (Z = -0.166; *p *> 0.05) was observed for the prediction of behaviour whereas for the prediction of intention, studies where the information was presented and the psychometric qualities were good, a higher frequency-weighted mean R^2 ^value was observed (Z = -10.925; *p *< 0.0001). Finally, concerning the prediction of behaviour, a better frequency-weighted mean R^2 ^was observed when behaviour was self-reported compared to objectively assessed (Z = 9.521; *p *< 0.0001). In this latter case, the frequency-weighted mean R^2 ^value for the prediction of behaviour varied according to the level of correspondence between intention and behaviour; a better prediction of behaviour was observed when the level of correspondence was appropriate (Z = -7.993; *p *< 0.0001).

**Table 4:** Model efficacy to predict healthcare professionals' behaviours and intentions according to the methodological qualities of the studies

Characteristic of the studies	Number of participants (studies)	Frequency- weighted mean R^2^
Prediction of behaviour		
Sample size		
- N < 150	833 (12)	0.22
- N ≥ 150	1 279 (3)	0.38
Psychometric quality		
- No information/poor values	1 119 (7)	0.31
- Complete information/good values	993 (8)	0.32
Behavioural measure		
- Self-report	1 286 (4)	0.44
- Objective	826 (11)	0.13
Level of correspondence for intention-behaviour*		
- Poor/unclear	546 (6)	0.10
- Good	1 566 (9)	0.39
Prediction of intention		
Sample size		
- N < 150	3 187 (34)	0.50
- N ≥ 150	11 799 (30)	0.61
Psychometric quality		
- No information/poor values	3 112 (15)	0.47
- Complete information/good values	11 874 (49)	0.62

* The intention-behaviour correspondence was good for all self-reported measurements

Note: Because there were missing data in few publications, total differs from 16 and 72 studies for the behaviour and intention, respectively.

## Discussion

The present study examined the efficacy of studies based on social cognitive theories in explaining intention and predicting the clinical behaviour of healthcare professionals. By means of a systematic review, the overall efficacy was evaluated and the effect of factors that could affect the efficacy of prediction was also examined. Overall, the efficacy of prediction of behaviour was equivalent to values reported in several meta-analyses of the TPB, the most widely used social cognition model of health behaviour. For instance, between 25.6% and 34% of explained variance in behaviour was reported for applications of the TPB [9,99]. The current frequency-weighted mean R^2 ^of 0.31 for the prediction of healthcare professional' behaviours compares very favourably to these figures. Regarding the prediction of intention, however, the value observed in the present study (59% explained variance) was higher that the values reported for applications of the TPB (33.7% in Conner and Sparks [9], and 40% in Godin and Kok [99]). A possible explanation for this is that the present review was not limited to the TPB. Other theories were investigated and consequently variables other than those identified in the TPB were considered in the prediction. For instance, role beliefs and moral norm are important variables in Triandis' theory that emerged as substantial determinants of intention.

This systematic review also showed that a number of factors affect the efficacy of prediction of intention/behaviour. On this regard, type of health professionals and behaviour categories, sample size, psychometric qualities, method for assessing behaviour, level of correspondence between the operational definitions of intention and behaviour required special attention.

Variations in the efficacy of prediction of intention and behaviour were observed between types of healthcare professionals. In the prediction of behaviour, the best predictive models were observed for healthcare professionals other than physicians and nurses, whereas the best prediction of intention was observed among the nurse samples. Similarly, important variations in explained variance of professionals' behaviours and intentions were observed between behavioural categories. It is not clear what underlies these variations in efficacy of prediction, but one possible explanation could be the nature of the behaviour to be performed and the context of practice. This was particularly evident in prospective studies among physician samples, in which these two elements were defined more vaguely probably because the clinical practice of physician is more difficult to define accurately. This interpretation is further supported by our observation that the operational definitions of intention in terms of action and context for the prediction of behaviour were generally more precise in other healthcare professional samples compared with the studies of physician samples. Given the complexity of clinical-related behaviours, and particularly for diagnostics and treatment decisions, behaviour adoption could be modulated by several aspects of the context, such as patients' acceptability or preference for a given treatment, characteristics of the health problems, new versus usual patients, patients with multiple symptoms, antecedents or counter indications for a given type of medication, etc. Consequently, the accuracy of intention to predict future behaviour is reduced. Obviously, further research should pay more attention not only to the definition of the targeted behaviour, but also to its context of realization. As such, the use of vignettes could be a useful avenue to define more specifically the context of behavioural performance. For instance, Harrell and Bennett [22] successfully used a vignette to predict prescribing behaviour among a physician sample. They were able to explain 26.8% of variance in a behaviour assessed objectively. Thus, the use of vignettes could help healthcare professionals to better define the context of behavioural performance and formulate their intention more accurately. Consequently, the efficacy of social cognitive theories to understand healthcare professionals' behaviour could be improved and the findings could be more appropriate to inform future interventions.

Other methodological aspects were also scrutinized in the present review, and obviously they require special attention given their significant impact on the efficacy to explain intention and predict behaviour. For instance, when an objective measure of behaviour was obtained, the efficacy of prediction was much lower than when self-report measures were used. This observation is congruent with the results reported by Armitage and Conner [100] for the prediction of behaviour. They observed a significant difference between the proportion of variance explained when behaviours were observed (R^2 ^= 0.20) compared to self-reported (R^2 ^= 0.31). It can be argued that the objective assessment of behaviour is less subject to several biases (including reporting bias) than self-reports and consequently is more accurate in measurement. However, the majority of the studies using an objective measure of behaviour did not comply with the principle of correspondence between intention and behaviour, as recommended by Fishbein and Ajzen [13] (and acknowledged by most theorists in social psychology). Again, the main discrepancies were noted for the action and context dimensions; that is, the action and context mentioned in the statement of intention did not fully correspond to the behavioural measured obtained. For example, in the study by Sauls [30], the intention of intra-partum nurses was formulated with respect to several specific actions related to professional labour support during childbirth. However, the measure obtained as the behavioural outcome was the patients' length of labour. This resulted in a lack of correspondence between what was measured and what was intended. In summary, one cannot eliminate flaws in methods as an explanation for the poor efficacy in prediction when objective measures were taken. This appears to be an important point that will require further investigation.

Another methodological aspect affecting the efficacy in prediction is sample size. A lower prediction was observed among studies with smaller sample sizes. This observation supports the thorough analysis by Rashidian and colleagues [18] who estimated the sample size that should be used for a random survey of prescribing intention and actual prescribing for a study based on the TPB. Based on the variance inflation factor method, they suggested that a sample size of 148 should be recruited. This suggests that studies of healthcare professionals' behaviours should be planned in order to recruit the appropriate number of participants. If this condition is not met, the potential to obtain an efficient predictive model is reduced.

The results also indicated that good psychometric values are essential to explain a greater proportion of the intention variance. It has been documented that the reliability of a scale affects its predictive power; poor prediction results from poor reliability [101]. This effect was not observed for the prediction of behaviour, but the number of studies was relatively small compared to the number of studies available for the analysis of intention.

To guide the analysis of the variables measured to predict intention and behaviour, we used the comprehensive approach suggested by Michie and colleagues [8]. This approach was found to be very useful to capture most of the dimensions that were used to study healthcare professionals' behaviours. Notwithstanding the quality of their classification, we added two categories to their method: moral norm and habit/past behaviour. This decision is supported by the finding that moral norm as a single construct was found to be a significant determinant of intention seven out of ten times when assessed. It is also likely that with the addition of studies on the prediction of behaviour, the importance of past behaviour/habit will progressively emerge. This anticipated result is based on the observations of Verplanken and Woods [102] who demonstrated that habitual behaviour performed in a stable context is more difficult to change. Given that many of the behaviours performed by healthcare professionals could be categorized as habitual because they are typically performed in a stable context, this aspect should be documented in future studies. Unfortunately, at this time, it is not possible to verify this assumption as the number of applications was not sufficient.

**Figure 2:**
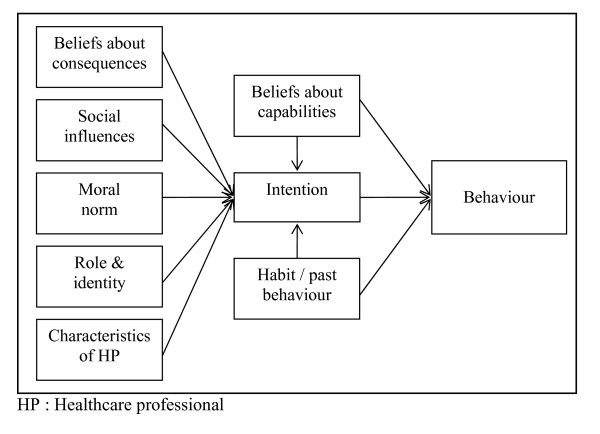
Hypothesized theoretical framework for the study of healthcare professionals' behaviour and intention.

One of the key questions addressed by this review is which theory or theoretical construct is the most relevant for the study of healthcare professionals' behaviours. Our results suggest that the TPB is an appropriate theory to predict behaviour, whereas Triandis' theory better captures the dynamic underlying intention. Indeed, the two categories of variables predicting behaviour most often (when assessed) were intention and beliefs about capabilities. This latter category includes the concept of perceived behavioural control, one of the TPB determinants of behaviour alongside intention. Concerning the determinants of intention, the situation is more complex, because five categories of variables significantly contributed to its prediction (*i.e.*, most of the time when assessed). These categories of variables were: beliefs about capabilities, beliefs about consequences; moral norm; social influences; and role and identity. According to Triandis' theory, these variables would correspond to facilitating factors, cognitive attitude, moral norm, social norm, and role beliefs, respectively. Finally, even if habit did not emerge as one of the important determinants predicting behaviour, it has been added because according to Weinstein [103] its effect should be controlled in longitudinal studies. Thus, direct links with both intention and behaviour are anticipated. Interestingly, this variable is also included in Triandis' theory. We have illustrated the interrelationship of these variables in the prediction of intention and behaviour in Figure 2. We do not imply that other factors are not important, but it appears from our analysis, that the integration of the variables presented in Figure 2 summarizes the majority of our observations.

A number of limitations should be noted. First, a limited number of studies predicting behaviour were identified. It appears that most of the effort invested was concerned with understanding intention. Not much attention has been given to prospective studies aimed at predicting behaviour. More studies of behaviour prediction are therefore strongly needed to understand which factors underlie the cognitive process of decision-making in clinical-related behaviours. Second, in our analysis of the efficacy of prediction, we did not control for the number of variables included in the predictive models. We acknowledge that this might have inflated the relative performance of some theories over more parsimonious ones.

## Conclusion

In conclusion, this study was the first systematic review aimed at investigating applications of different social cognitive theories for the study of clinical-related behaviours of health professionals. This is an important first step in identifying variables explaining intention and predicting clinical-related behaviours. Nonetheless, a number of methodological factors were identified as potential moderators of the efficacy in prediction of studies based on social cognitive theories. Future studies should take into consideration methodological aspects in order to contribute to the development of a significant corpus of data on the clinical behaviours of healthcare professionals. In particular, special care should be given to better define the context of behaviour performance. In addition, we noted that there is an important lack of prospective studies predicting healthcare professionals' clinical-related behaviours; only 16 studies were identified. Thus, there is an urgent need of additional prospective studies based on sound theoretical frameworks. We hope that the information provided in this review of the scientific literature will be useful to researchers in the planning of studies that may lead to improved strategies to change healthcare professionals' behaviours.

## Competing interests

The authors declare that they have no competing interests.

## Authors' contributions

GG, JG and ME conceptualized the review and had regular discussion on this topic in KT ICEBERG meetings. ABG coordinated and performed the acquisition of data as well as the statistical analysis. GG helped conduct the data analysis and interpretation. GG and ABG drafted the manuscript. ME and JG provided critical review on all parts of the manuscript. All authors approved the final version of the manuscript.

## Supplementary Material

Additional file 1 **The search strategy**. This table describes the literature search strategy used for this review. Click here for file

Additional file 2 **Prospective studies aimed at predicting health professionals' behaviour**. This table is the synthesis of data abstraction for studies aimed at predicting healthcare professionals' behaviours. Click here for file

Additional file 3 **Studies aimed at predicting health professionals' intentions**. This table is the synthesis of data abstraction for studies aimed at predicting healthcare professionals' intentions. Click here for file

Additional file 4 **Classification of variables**. This table describes the domains of the variables extracted for the review. Click here for file
