# Supplementary material for: Healthcare professionals' intentions and behaviours: A systematic review of studies based on social cognitive theories
Source: Implement Sci. 2008 Jul 16;3:36. doi: 10.1186/1748-5908-3-36 (PMC2507717; doi:10.1186/1748-5908-3-36)
Supplement: Additional file 1 — The search strategy. This table describes the literature search strategy used for this review. [file 1748-5908-3-36-S1.pdf]

Additional file 1 – The search strategy

**MESH terms, keywords or descriptors**	**Type of healthcare professionals**
Behaviour OR intention	Physician
Planned behaviour	General practitioner
Social cognitive theory	Different specialities of medicine:
Bandura's theory	anaesthesiologist; geriatrician; gynaecologist;
Triandis' theory	haematologist; hospitalist; internist; nephrologist;
Psychosocial theory	neurologist; obstetrician; oncologist; optometrist;
	paediatrician; psychiatrist; radiologist; resident;
	surgeon; urologist
	Chiropractor
	Dentist
	Dietician
	Health professional
	Kinesiologist
	Mental health professional
	Nurse
	Pharmacist
	Physical therapist
	Psychologist
