# Supplementary material for: Healthcare professionals' intentions and behaviours: A systematic review of studies based on social cognitive theories
Source: Implement Sci. 2008 Jul 16;3:36. doi: 10.1186/1748-5908-3-36 (PMC2507717; doi:10.1186/1748-5908-3-36)
Supplement: Additional file 2 — Prospective studies aimed at predicting health professionals' behaviour. This table is the synthesis of data abstraction for studies aimed at predicting healthcare professionals' behaviours. [file 1748-5908-3-36-S2.pdf]

Additional file 2 – Prospective studies aimed at predicting health professionals' behaviour

Study	1. Type of participants<br>2. Sample size	1. Theoretical framework<br>2. Behaviour studied	Measure of behaviour	Level of correspondence between intention and behaviour	Final model	R^2
Bernaix [26]	1. Maternal-newborn nurses<br>2. 49	1. TRA<br>2. Providing support for breastfeeding	Perception of support by mothers (OBJ)	Good	Attitude<br>Knowledge<br>Ethnicity<br>Education	0.56
Eccles et al. [20]	1. General practitioners<br>2. 230	1. OLT<br>2. Managing infections without antibiotics	Total number of antibiotic prescriptions (OBJ)	Poor	Habitual behaviour	0.063
Farris & Schopflocher [33]	1. Community pharmacists<br>2. 182	1. SCT<br>2. Pharmaceutical care	Provided or not pharmaceutical care (SR)	Good	Self-efficacy	O/S
Gilomen-Study [21]	1.Physicians<br>2. 45	1.TPB<br>2. Prescribing	Coded prescription data (OBJ)	Poor	Attitude	0.15

Godin et al. [27]	1. Nurses<br>2. 105	1. TPB<br>2. Adherence to universal precautions for venipuncture	Number of time they adhered to universal precautions (SR)	Good	Intention<br>PBC	0.28
Harrell & Bennett [22]	1. Physicians<br>2. 93	1. TRA<br>2. Prescribing	Physician panel data (OBJ)	Poor	Intention	0.27
Hoppe [31]	1. Nurses<br>2. 132	1. TPB<br>2. Weight loss management	Number of time they raised the issue of weight loss (SR)	Good	Intention<br>Self-efficacy	0.46
Lambert et al. [23]	1. Physicians<br>2. 19	1. TRA<br>2. Antibiotic prescribing	Antibiotic prescribing data (OBJ)	Poor	-	0.0049
Mason [34]	1. Pharmacists<br>2. 40	1. TRA<br>2. Counseling	Direct observation (OBJ)	Good	Attitude<br>Subjective norm	0.33
Maue et al. [24]	1. Providers<br>2. 33	1. TPB<br>2. Compliance with practice guidelines	Medical record audits (OBJ)	Good	Attitude	0.0076
Millstein [25]	1. Primary care	1. TPB	Percentage of	Good	Intention	0.40

	physicians<br>2. 765	2. Providing education about STD-HIV	adolescents being educated (SR)		Subjective norm<br>PBC	
O'Boyle et al. [28]	1. Nurses in critical & post critical care units<br>2. 120	1. TPB<br>2. Adherence to hand hygiene recommendations	2-hour observation period (OBJ)	Poor	Intensity of activity in the unit	0.12
Quinn [32]	1. Nurses<br>2. 50	1. TRA<br>2. Documentation	Analysis of patient records (OBJ)	Good	-	0.01
Renfroe et al. [29]	1. Nurses<br>2. 108	1. TRA<br>2. Documentation	Analysis of patient's chart (0.71)[a] (OBJ)	Good	Intention	0.15
Sauls [30]	1. Intrapartum nurses<br>2. 39	1. TPB<br>2. Labour support	Length of labour (OBJ)	Poor	-	-0.05
Wilson [35]	1. School psychologists<br>2. 284	1. TPB<br>2. Using a consultation model	Use of consultation model (SR)	Good	Intention<br>PBC	0.58

OBJ: Objective assessment of behaviour; OLT: Operant Learning Theory; O/S Other statistics; SCT: Social Cognitive Theory; SR Self-reported behaviour; TPB: Theory of Planned Behaviour; TRA: Theory of Reasoned Action.

[a] KK-20 reliability coefficient
