# Supplementary material for: Healthcare professionals' intentions and behaviours: A systematic review of studies based on social cognitive theories
Source: Implement Sci. 2008 Jul 16;3:36. doi: 10.1186/1748-5908-3-36 (PMC2507717; doi:10.1186/1748-5908-3-36)
Supplement: Additional file 3 — Studies aimed at predicting health professionals' intentions. This table is the synthesis of data abstraction for studies aimed at predicting healthcare professionals' intentions. [file 1748-5908-3-36-S3.pdf]

Additional file 3 – Studies aimed at predicting health professionals' intentions

Study	1. Type of participants<br>2. Sample size	1. Theoretical framework<br>2. Behaviour studied	Measure of intention<br>Nb of items (PQ) [a]	Final model	R^2
Beatty & Beatty [36]	1. Anaesthetists<br>2. 114	1. TPB<br>2. Violating safety guidelines (not performing a pre-operative visit)	1	Attitude<br>Subjective norm<br>PBC<br>Habit	0.45
Bernaix [26]	1. Maternal-newborn nurses<br>2. 49	1. TRA<br>2. Support of breastfeeding mothers	3 (0.93)	Attitude<br>Subjective norm<br>Ethnicity<br>Level of education	0.72
Bolman et al. [60]	1. Nurses<br>2. 85	1. ASE<br>2. Continuing to use a smoking cessation protocol	1	Perceived complexity<br>Perceived advantages	0.38
Bonnetti et al. [37]	1. General practitioners<br>2. 214	1. TPB<br>2. Referring for X-ray	3 (0.82)	Attitude<br>PBC	0.31
Bonnetti et al. [83]	1. General dental	1. OLT	3 (0.73)	Anticipated consequences	0.43

	professionals<br>2. 214	2. Taking intra-oral radiographs		Evidence of habit	
Bunce & Birdi [38]	1a. Lower grade doctors<br>1b. Senior doctors<br>2b. 48<br>2a. 70	1. TPB<br>2. Requesting hospital autopsies	1	a. Attitude<br>Subjective norm<br>PBC<br>b. Attitude<br>Subjective norm<br>Past behaviour	0.43<br>0.65
Busha [39]	1. Family physicians<br>2. 100	1. TPB<br>2. Educating about pregnancy prevention (among males 15 years old)	1	Subjective norm<br>PBC<br>Perceived benefices	0.47
Chau & Hu [40]	1. Physicians<br>2. 408	1. TAM<br>2. Using telemedicine technology	2 (0.77)	Attitude<br>Perceived usefulness	0.40
Conner & Heywood-Everett [41]	1. General practitioners<br>2. 65	1. TPB<br>2. Addressing mental health problems (Asian patients)	1	Subjective norm (SN)<br>Motivation to comply (MC)	0.32

				Past behaviour<br>SN X MC	
Crawford [61]	1. Critical care nurses<br>2. 225	1. TPB<br>2. Involvement with families in the intensive care unit	3 (0.85)	Attitude<br>Subjective norm<br>Internal factors<br>Workload<br>Baccalaureate degree	0.56
Croteau & Vieru [42]	1a. Physicians in urban areas<br>1b. Physicians in rural areas<br>2a. 85<br>2b. 42	1. TAM<br>2. Telemedicine adoption	4 (-)	a. Perceived ease of use<br>Perceived usefulness<br>Perceived voluntariness<br>b. Perceived usefulness	0.60<br><br><br>0.48
Daneault et al. [84]	1. Nurses & dieticians<br>2. 151	1. TPB<br>2. Recommending breastfeeding	2^b	PBC<br>Perceived professional norm	0.69
Dilorio [62]	1. Neuroscience nurses<br>2. 368	1. TPB<br>2. Care for persons with	11 (0.86)	PBC	0.16

		HIV+/AIDS			
Eccles et al. [20]	1. General practitioners<br>2. 230	1. OLT<br>2. Managing infections without antibiotics	3 (0.68)	Anticipated consequences<br>Habitual behaviour	0.43
Edwards et al. [63]	1. Nurses<br>2. 446	1. TPB<br>2. Administering opioids for pain relief	3 (0.79)	Attitude<br>Behavioural beliefs<br>Subjective norm<br>PBC	0.40
Farris & Kirkking [85]	1. Community pharmacists<br>2. 169	1. TRA (TT)<br>2. Pharmaceutical care	2 (0.65)	Social norm<br>Frequency of past trying	0.17
Faulker & Biddle [86]	1. Mental health professionals<br>2. 394	1. TPB<br>2. Promoting physical activity	2 (0.84)	Attitude<br>Subjective norm<br>PBC<br>Past stage of change	0.71
Feug & Wu [64]	1. Nurses<br>2. 1362	1. TPB<br>2. Reporting child abuse (severe cases)	4 (0.62)	Attitude<br>Subjective norm<br>PBC	0.91

				Knowledge	
Fisher & Pathak [43]	1. Physicians<br>2. 93	1. TRA<br>2. Using pharmacists as drug information consultants	1	Attitude	0.23
Foy et al. [87]	1. Mental health professionals<br>2. 373	1. TPB<br>2. Disclosing a diagnostic of dementia (Exploring what patients already knows)	2 (0.85)	Attitude<br>Emotional attitude<br>Subjective norm<br>PBC	0.29
Foy et al. [44]	1. Gynaecologists<br>2. 151	1. TPB<br>2. Guideline compliance (Assessing an appointment within 5 days of referral)	3 (>0.70)	Subjective norm	0.27
Gagnon et al. [46]	1. General practitioners<br>2. 519	1. TIB<br>2. Acceptance of telemedicine	3 (0.84)	Normative factors<br>Self identity	0.81
Gagnon et al. [45]	1a. Ophthalmologists<br>1b. Orthopaedic surgeons	1. TIB<br>2. Using the Health Technology Assessment recommendations	3 (0.85)	a. Attitudinal beliefs<br>Personal normative beliefs	0.87

	2a. 35<br>2b. 61			Social normative beliefs<br>b. Facilitating conditions<br>Personal normative beliefs<br>Habit	0.65
Gaither et al. [47]	1. Physicians<br>2. 87	1. TRA<br>2. Utilization of drug information sources (physicians, colleagues)	1	Attitude	0.14
Gilomen-Study [21]	1. Physicians<br>2. 45	1. TPB<br>2. Prescribing	1	Attitude	0.59
Godin et al. [48]	1a. General practitioners<br>1b. Residents<br>2a. 201<br>2b. 144	1. TPB<br>2. Recommending complementary and alternative medicine	2[c]	a. Attitude<br>PBC<br>Moral norm<br>Descriptive norm<br>b. Attitude<br>PBC<br>Moral norm	0.83<br>0.66

Godin et al. [49]	1. Physicians<br>2. 852	1. TPB<br>2. Examining an HIV+/AIDS patient	1	Attitude<br>Perceived norm<br>Self-efficacy<br>Tests<br>Sex X age<br>Risk X attitude	0.50
Godin et al.[95]	1. Dentists<br>2. 771	1. TPB<br>2. Providing dental care to HIV+/AIDS patients	2 (0.90)	PBC<br>Personal normative beliefs<br>Habit	0.71
Godin et al. [50]	1. Physicians<br>2. 667	1. TPB<br>2. Wearing gloves	1	Attitude<br>PBC<br>Perceived social norm<br>Perceived risk<br>Habit<br>Age	O/S
Godin et al. [27]	1. Nurses	1. TPB	4 (0.82)	Barriers	0.68

	2. 156	2. Adherence to universal precautions for venipuncture		Subjective norm<br>Personal normative beliefs	
Herbert & Benbasat [65]	1. Nurses<br>2. 151	1. TRA<br>2. Adoption of information technology	4 (-)	Relative advantages<br>Result demonstrability<br>Compatibility<br>Normative beliefs	0.77
Herbert et al. [88]	1. Pharmacists<br>2. 203	1. TPB<br>2. Providing therapy management services	6 (0.88)	Attitude<br>Subjective norm<br>PBC	0.63
Hoppe [31]	1. Nurses<br>2. 102	1. TPB<br>2. Obesity management	6 (0.94)	Attitude<br>Subjective norm<br>Self-efficacy<br>Self-identity<br>Past behaviour	0.60
Hu et al. [51]	1. Physicians<br>2. 408	1. TAM<br>2. Adoption of telemedicine	6 (0.86)	Attitude<br>Perceived usefulness	0.44
Jenner et al. [89]	1. Health professionals	1. TPB	4 (0.78)	Attitude	O/S

	2. 100	2. Hand hygiene practice		Personal responsibility	
Jones et al. [52]	1. Oncologists<br>2. 281	1. TPB<br>2. Recommending to exercise	1	Attitude<br>Subjective norm<br>PBC	0.22
Jurgens [66]	1. Nurses<br>2. 149	1. TPB<br>2. administering morphine for postoperative pain	2 (0.69)	Attitude<br>PBC<br>Control beliefs	0.63
Kittisopee [90]	1. Pharmacists<br>2. 269	1. TPB<br>2. Pharmaceutical care	3 (0.76)	Attitude<br>Subjective norm<br>PBC<br>Agreeableness	0.78
Klaybor [91]	1. Social workers<br>2. 249	1. TPB<br>2. Using the DSM-IV for treatment planning	2 (-)	Attitude<br>Norm<br>PBC	0.25
Lambert et al. [23]	1. Physicians<br>2. 19	1. TRA<br>2. Prescribing	-	Attitude<br>Subjective norm	0.62
Laschinger &	1. Nurses	1. TRA	1	Attitude	0.19

Goldenberg [67]	2. 141	2. Care for HIV+ patients			
Laschinger et al. [68]	1. Community health nurses<br>2. 145	1. TRA<br>2. Care for HIV+ patients	1	Attitude	0.19
Lavoie et al. [69]	1. Nurses<br>2. 119	1. TPB<br>2. Selecting a given level of care	4 (0.82)	Attitude (cognitive)<br>Perceived professional norm<br>Living will	0.86
Légaré et al. [53]	1a. General practitioners (France)<br>1b. Gynaecologists (France)<br>1c. General practitioners (Québec)<br>1d. Gynaecologists (Québec)<br>2a. 419<br>2b. 577<br>2c. 333	1. TPB<br>2. Prescribing hormone replacement therapy	2 (0.84)[d]	a. Role beliefs<br>PBC<br>Control beliefs<br>Cessation of menses<br>b. Role beliefs<br>Moral norm<br>Control beliefs<br>Lipid profile<br>c. Moral norm<br>Control beliefs	O/S

	2d. 129			Cytology<br>Hormonal dosage<br>d. Moral norm<br>Control beliefs	
Légaré et al. [54]	1. Physicians and residents<br>2. 121	1. TPB<br>2. Screening for decisional conflict	3 (0.79)	Attitude<br>Subjective norm<br>PBC<br>Clinical site	0.70
Levin et al. [92]	1. Nurses & medical laboratory workers<br>2. 527	1. TPB<br>2. Wearing gloves	1	Attitude<br>PBC<br>Perceived risk	0.73
Liabsuetrakul et al. [55]	1. Obstetricians<br>2. 50	1. TPB<br>2. Administration of antibiotic after cord clamping	-[e]	Subjective norm	0.25
Limbert & Lamb [56]	1a. Hospital doctors<br>1b. Hospital doctors	1. TPB<br>2a.Compliance with asthma guidelines	a.1<br>b. 3 (-)	a. Attitude<br>Subjective norm<br>PBC	0.58

	2a. 223<br>2b. 214	2b.Compliance with antibiotic guidelines		b. Attitude<br>Subjective norm<br>PBC	0.52
Maue et al. [24]	1. Physicians & nurse practitioners<br>2. 60	1. TPB<br>2. Compliance with guidelines	2 (>0.60)	Attitude<br>PBC<br>Level of difficulty	0.68
McKinlay et al. [70]	1. Nurses<br>2. 74	1. TRA<br>2. Care of self-poisoning patients	2 (-)	Attitude<br>Subjective norm	0.66
Milstein [25]	1. Primary care physicians<br>2. 765	1. TPB<br>2. Providing education about STD-HIV	1	Attitude<br>Social norm<br>PBC	0.27
Nash et al. [71]	1. Nurses<br>2. 100	1. TRA<br>2. Pain assessment	2 (-)	PBC	0.21
O'Boyle et al. [28]	1. Nurses<br>2. 120	1. TPB<br>2. Adherence to hand hygiene recommendations	5 (0.74)	Subjective norm<br>Control beliefs	0.56
Payant [72]	1. Nurses	1. TPB	3 (0.91)	Subjective norm	0.55

	2. 97	2. Providing continuous labour support		PBC<br>Labour support courses	
Pessoa-silva et al. [93]	1. Healthcare workers<br>2. 61	1. TPB<br>2. Hand hygiene	12 (0.81)	Perceived difficulties<br>Subjective norm	O/S
Puffer & Rashidian [73]	1. Nurses<br>2. 48	1. TPB<br>2. Using clinical guidelines	2 (0.62)	Attitude<br>PBC	0.40
Renfroe et al. [29]	1. Nurses<br>2. 108	1. TRA<br>2. Documentation	2 (-)	Attitude<br>Subjective norm	0.46
Ryu et al. [57]	2. Physicians<br>3. 286	1. TPB<br>2. Knowledge sharing	5 (0.91)	Attitude<br>Subjective norm<br>PBC	O/S
Sable et al. [58]	1. Primary care physicians<br>2. 96	1. TRA<br>2. Prescribing	5 (-)	Attitude<br>Subjective norm	N/A
Sauls [30]	1. Intrapartum nurses<br>2. 39	1. TPB<br>2. Providing labour support	27 (-)	Attitude<br>Subjective norm	0.67
Schottle [74]	1. Obstetrical nurses	1. TPB	1	Attitude	0.52

	2. 26	2. Collaborating with midwifes		Subjective norm	
Tessaro & Highriter [75]	1. Nurses<br>2. 311	1. TRA<br>2. Working with HIV+/AIDS patients	4 (0.86)	Attitude<br>Subjective norm<br>Prior behaviour<br>Ever cared for patients with HIV/AIDS patients<br>Number of years in public health<br>Supervisory position	N/A
Vermette & Godin [76]	1. Nurses<br>2. 102	1. TPB<br>2. Providing care at home to AIDS or leukaemia patients	3 (0.83)	Attitude<br>Personal normative beliefs<br>PBC<br>Homophobia	0.48
Walker et al. [59]	1. General practitioners<br>2. 126	1. TPB<br>2. Prescribing	1	PBC<br>Control beliefs<br>Past behaviour	0.62
Walker et al. [94]	1. Community pharmacists	1. TPB	2 (0.85)	Attitude	0.14

	2. 76	2. Treatment of vaginal candidiasis with non-prescription medicines			
Wallace et al. [77]	1. Nurses<br>2. 85	1. TRA<br>2. Providing oral care to patients receiving chemotherapy	4 (0.72)	Attitude<br>Subjective norm	0.39
Walsh et al. [78]	1.Pediatric nurses<br>2. 51	1. TPB<br>2. Fever management	2 (0.73)	Beliefs-based attitude<br>Subjective norm<br>Indirect control	0.25
Waltman & Corless [79]	1. Nurses<br>2. 372	1. TRA<br>2. Care of the dying	12 (-)	Attitude<br>Normative beliefs	0.72
Watson & Myers [80]	1. Nurses<br>2. 103	1. TPB<br>2. Clinical glove use	3 (0.75)	Attitude<br>PBC<br>Barriers	0.45
Werner & Mendelsson [81]	1. Nurses<br>2. 303	1. TRA<br>2. Using physical restraints with older people	4 (0.70)	Attitude<br>Subjective norm<br>Perceived moral obligation	0.48

Whitby et al. [82]	1. Nurses<br>2. 754	1. TPB<br>2. Elective in-hospital hand washing	-	Attitude<br>Subjective norm<br>Perceived peer behaviour<br>Effort required<br>Elective community behaviour	0.64
Wilson [35]	1. School psychologists<br>2. 284	1. TPB<br>2. Using a consultation model	10 (0.93)	Attitude<br>Subjective norm<br>PBC	0.46

ASE: Attitude, Social and Self-Efficacy Model; N/A: Not available; PBC: Perceived behavioral control; PQ: Psychometric qualities; OLT: Operant Learning Theory; O/S: Other statistics; SCT: Social Cognitive Theory; TAM: Technology Acceptance Model; TIB: Theory of Interpersonal Behaviour; TPB: Theory of Planned Behaviour; TRA: Theory of Reasoned Action; TT: Theory of Trying.

[a] Cronbach's Alpha coefficient (α)

[b] Alpha coefficients were considered sufficient for all variables including intention

[c] α (0.77 à 0.88) or r (0.55 à .71) for all constructs including intention

[d] Pearson correlation coefficient

[e] Alpha coefficients ranged from 0.64 to 0.85 for all variables including intention
