# Supplementary material for: Healthcare professionals' intentions and behaviours: A systematic review of studies based on social cognitive theories
Source: Implement Sci. 2008 Jul 16;3:36. doi: 10.1186/1748-5908-3-36 (PMC2507717; doi:10.1186/1748-5908-3-36)
Supplement: Additional file 4 — Classification of variables. This table describes the domains of the variables extracted for the review. [file 1748-5908-3-36-S4.pdf]

Additional file 4 – Classification of variables

Domains	Constructs
Knowledge	Knowledge, perceived similarity between guidelines and current practice
Social / professional role and identity	Competence and professionalism, image, personal responsibility, professional norm, role beliefs, self-identity,
Moral norm	Moral norm, personal normative beliefs
Beliefs about capabilities	Compatibility, computer avoidance, continuing education, control beliefs, decision difficulty, ease to use, likelihood of a clinical situation arising, likelihood of using the pharmacist in a situation, organizational barriers, perceived barriers, perceived behavioural control, perceived complexity, perceived effort required, perceived facilitating conditions/factors, perceived ease to use, perceived level of difficulty, potential work-related exposure, self-efficacy, time, voluntarism, workload, personal & professional interactions
Beliefs about consequences	Anticipated consequences, attitude, behavioural beliefs, external factors (not be adequately reimbursed & time-consuming), pain beliefs, perceived advantages, perceived benefices, perceived

	consequences, perceived moral obligation, perceived risk, perceived utility, results demonstrability,
Motivation and goals	Intention
Environmental context and resources	Clinical site, situational support, intensity of activity in the nursing unit, environment, settings, adequacy of resources, practice pattern-related, examinations performed,
Social influences	administrative support, cosmopolitan perspective, descriptive norm, family factors, modeling, normative beliefs, normative factors, perceived peer behaviour social norm, social participation, social support, subjective norm, team support
Emotion	Affect, anxiety, emotional attitude, emotional response, experienced consequences, homophobia, reluctance to disclose uncertainty
Past behaviour / habit	actual behaviour, community hand washing, evidence of habitual behaviour, habit, in-hospital hand washing, experience, negative experience, positive experience
Characteristics of health professionals	Socio-demographic, personal breastfeeding histories, personality (extraversion, neuroticism, agreeableness, openness, conscientiousness)

Adapted from Michie et al. (2005)
